# Supplementary material for: Personalised Medicine for Colorectal Cancer Using Mechanism-Based Machine Learning Models
Source: Int J Mol Sci. 2021 Sep 15;22(18):9970. doi: 10.3390/ijms22189970 (PMC8467693; doi:10.3390/ijms22189970)
Supplement: Supplementary file 1 [file ijms-22-09970-s001.zip › ijms-1339236-supplementary/Supplementary File/Supplemental 9_List of Abbreviations.pdf]

Abbreviations

Colorectal Cancer (CRC)

Consensus Molecular Subtypes (CMS)

signal transduction networks (STN's)

TGF-β (Transforming growth factor beta)

PI3K/Akt (phosphatidylinositol 3-kinase / protein kinase B)

TP53 (tumor protein P53)

MAPK (microtubule associated protein kinase)

Cell Cycle (cell-division cycle)

mTOR (Mammalian target of rapamycin)

WNT (Wingless-related integration site)

DKK (Dickkopf)

APC (adenomatous polyposis coli)

sFRP (secreted frizzled-related protein)

FZD (frizzled)

LRP5/6 (low density lipoprotein receptor-related protein 5/6)

GSK3β (Glycogen Synthase Kinase 3 Beta)

PROGENy (Pathway RespOnsive GENes for activity)

TCGA (The Cancer Genome Atlas)

mRNA (messenger ribonucleic acid)

DVL3 (Dishevelled Segment Polarity Protein 3)

VANGL2 (Vang-like protein 2)

CER1 (Cerberus 1)

TCF7L1 (Transcription Factor 7 Like 1)

CSNK2A1 (casein kinase 2 alpha 1)

LRP1 (low density lipoprotein receptor-related protein 1)

CSNK1A1 (casein kinase 1 alpha 1)

GPC4 (glypican 4)

PRKCA (protein kinase c alpha)

ROCK2 (rho associated coiled coil containing protein kinase 2)

NOTUM (notum, palmitoleoyl-protein carboxylesterase)

CTBP1 (c-terminal binding protein 1)

CTBP2 (c-terminal binding protein 2)

GPC4, PLCB4 (phospholipase c beta 4)

RAC1 (ras-related C3 botulinum toxin substrate 1)

CPTAC (Clinical Proteomic Tumor Analysis Consortium)
